# Treatment of upper aerodigestive tract cancers in England and its effect on survival

**DOI:** 10.1038/sj.bjc.6690695

**Published:** 1999-09

**Authors:** Dympna M Edwards, N W Johnson

**Affiliations:** Department of Oral and Maxillofacial Medicine and Pathology, Guy's, King's and Thomas' Schools of Medicine, Dentistry and Biomedical Sciences, King's Dental Institute, Caldecot Road, SE5 9RW, London, UK

**Keywords:** head and neck, cancer, surgery, radiotherapy, survival

## Abstract

The evidence base for head and neck cancers is low with relatively few randomized controlled trials of the two main treatments, surgery and radiotherapy. The aim of the study was to investigate the patterns of surgery and radiotherapy treatment for head and neck cancers in three large areas of England and to investigate their effects on survival. This was a retrospective study of 13 510 cases of head and neck cancers (ICD10: C00–C14, C30–C32) diagnosed and treated from 1984 to 1992 in England. We undertook multivariate analyses of survival using a step-wise Cox proportional hazard model and Kaplan–Meier analysis. There were regional variations in the treatments given to patients. Four in ten patients did not receive currently recommended treatments. In multivariate analyses treatment content and timing had an independent effect on survival. Better survival was associated with surgery for mouth cancers, radiotherapy for laryngeal cancers and combined treatment for pharyngeal cancers independent of tumour and demographic factors. Further research is needed to investigate the findings of this study through large randomized controlled trials and multi-centre audits. © 1999 Cancer Research Campaign


*British Journal of Cancer* (1999) **81**(2), 323–329
© 1999 Cancer Research Campaign
Article no. bjoc.1999.0695

### Treatment of upper aerodigestive tract cancers in England and its effect on survival

**Dymphna M Edwards and NW Johnson**

Department of Oral and Maxillofacial Medicine and Pathology, Guy's, King's and Thomas' Schools of Medicine, Dentistry and Biomedical Sciences, King's Dental Institute, Caldecot Road, London SE5 9RW, UK

**Summary** The evidence base for head and neck cancers is low with relatively few randomized controlled trials of the two main treatments, surgery and radiotherapy. The aim of the study was to investigate the patterns of surgery and radiotherapy treatment for head and neck cancers in three large areas of England and to investigate their effects on survival. This was a retrospective study of 13 510 cases of head and neck cancers (ICD10: C00–C14, C30–C32) diagnosed and treated from 1984 to 1992 in England. We undertook multivariate analyses of survival using a step-wise Cox proportional hazard model and Kaplan–Meier analysis. There were regional variations in the treatments given to patients. Four in ten patients did not receive currently recommended treatments. In multivariate analyses treatment content and timing had an independent effect on survival. Better survival was associated with surgery for mouth cancers, radiotherapy for laryngeal cancers and combined treatment for pharyngeal cancers independent of tumour and demographic factors. Further research is needed to investigate the findings of this study through large randomized controlled trials and multi-centre audits.

© 1999 Cancer Research Campaign

**Keywords:** head and neck; cancer; surgery; radiotherapy; survival

Despite advances in treatment, survival from upper aerodigestive tract cancers has not changed significantly on a population basis in Europe over the past 40 years (Berrino et al, 1995). In this study, upper aerodigestive tract (UAT) cancers refer to malignant neoplasms of the mouth, pharynx, larynx and associated structures (ICD10: C00–C14, C30–C32). They are mainly, but not exclusively, squamous cell carcinomas. Cancers of the thyroid, maxilla and mandible are excluded. UAT cancers are a significant and growing public health problem (MacFarlane et al, 1992). They are the third most common cancer in males world-wide and the seventh most common in females comprising 10.1% and 4.4% of cancer cases respectively (Parkin et al, 1993).

There is little population-based treatment information on UAT cancers available in the UK. Although a few individuals have developed audit databases (Woolgar, 1995; Worrall, 1995), in a 1996 survey of consultants treating head and neck cancers in the UK, only 4% of consultants reported using any standardized method of recording clinical information (Edwards et al, 1997). Over the past 50 years regional cancer registries in the UK have collected population-based information on cancers. They have limited treatment details, although there are differences in the type of information collected by each registry.

The major treatments for UAT cancers are surgery, radiotherapy and, to a lesser extent, chemotherapy. Surgical advances include the use of microvascular techniques, the radial forearm free flap (Soutar and McGregor, 1986; Vaughan et al, 1992; Vaughan and Brown, 1994) and percutaneous gastric feeding. The evidence base for treatments is low with most treatments being based on case series. Of the approximate 65 000 patients diagnosed with UAT cancers from 1984 to 1993 only 2273 (3.5%) patients were enrolled in randomized controlled trials of treatment registered with the United Kingdom Coordinating Committee on Cancer Research (UKCCCR, personal communication). Some analytical studies which compare the outcomes of different surgical treatments have been published, but these are generally non-randomized, retrospective observational studies with low numbers (Mendenhall et al, 1988; Weber et al, 1990; Davidson et al, 1991; Mitchell and Crighton, 1993; Fein et al, 1994; Nisi et al, 1998).

Radiotherapy fractionation protocols used in the UK have varied widely and been determined more by the equipment available than by evidence of effectiveness (Priestman et al, 1989). There has been a move towards combined surgical and radiotherapy treatment for advanced UAT cancers (Mendenhall et al, 1988; Glaholm, 1998; Nisi et al, 1998). Compared with single modality treatment combined surgery and radiotherapy has been found to improve outcomes for advanced tongue (Weber et al, 1990), pharyngeal (Bentzen et al, 1991) and laryngeal cancers (Vermund et al, 1990). Radiotherapy is routinely given post-operatively as it is associated with a lower recurrence rate than preoperatively (Tupchong, 1991). There is evidence from a number of studies that interruption in radiotherapy treatment and extension of treatment beyond 30 days because of interruptions reduce control rates for cancers of the oropharynx and larynx (Amdur et al, 1989; Slaladowski et al, 1994; Duncan et al, 1996; Wang et al, 1996).

There is some controversy about which treatments are appropriate for which cancers. Several studies have shown better functional and quality of life outcomes for patients treated with radiotherapy than for those treated with surgery for base of tongue cancers (Harrison et al, 1994; Moore et al, 1996; Zelefsky et al,

*Received 11 November 1998*
*Revised 24 February 1999*
*Accepted 12 April 1999*

*Correspondence to:* Dympna M Edwards, Department of Public Health, Liverpool Health Authority, 24 Pall Mall, Liverpool L3 6AL, UK

**Table 1** Recommended protocols and adherence to protocols

| Site | Recommended treatments | % Receiving recommended treatment | Recommended treatments | % Receiving recommended treatment |
|---|---|---|---|---|
| | **T1/T2** | **Extent of spread 1** | **T3/T4** | **Extent of spread 2–4** |
| Lip | Surgery or radiotherapy | 82 | Surgery and radiotherapy | 72 |
| Mouth | Surgery or radiotherapy | 71 | Surgery and radiotherapy | 35 |
| Oropharynx | Radiotherapy | 52 | Surgery and radiotherapy | 31 |
| Nasopharynx | Radiotherapy | 75 | Radiotherapy or surgery and radiotherapy | 96 |
| Larynx | Radiotherapy or surgery and radiotherapy | 71 | Surgery and radiotherapy | 77 |
| Salivary glands | Surgery | 38 | Surgery or surgery and radiotherapy | 81 |
| Total | | 67 | | 57 |

1996), buccopharyngeal cancers (Morton et al, 1984; Morton, 1997) and laryngeal cancers (Stewart et al, 1998), but others show better results for surgery (Fein et al, 1994). Although combined therapy may increase local control Rathmell et al (1991) found that patients with advanced UAT cancers treated with both surgery and radiotherapy had worse quality of life than those treated with radiotherapy alone.

There is anecdotal evidence of differences in treatment protocols between centres. Because of this a number of treatment guidelines have been developed. The British Association of Orolaryngologists–Head and Neck Surgeons have recently produced an extensive set of guidelines (1998). Treatment guidelines were considered by the British Association of Head and Neck Oncologists in an early draft of their publication on standards for head and neck cancer care (1998) and the British Association of Surgical Oncologists are in the process of producing guidelines for treatment. Surgical protocols have been developed by the German, Swiss and Austrian group DOSAK (Platz et al, 1986), there are joint guidelines by the American Society for Head and Neck Surgery and the Society of Head and Neck Surgeons (1996) and in a forthcoming publication Glaholm (1999) suggests recommended treatment for oral cancers. There is some agreement between the guidelines on recommended treatments. Table 1 outlines the common recommendations in the above guidelines. Recommendations usually focus on single modality treatment in small tumours and combined treatment in larger tumours. They recommend that radiotherapy be given within one month of surgery and delays in radiotherapy be avoided (Glaholm, 1999).

The Scottish Intercollegiate Guidelines Network (SIGN) grades the evidence on which guidelines are based from 1 to 4 (Grimshaw and Russell, 1993), 1 being the most reliable and 4 the least reliable. Most of the guidelines above on UAT treatment relating to surgery and radiotherapy treatment are based on level 3 and 4 evidence. The detail of the recommendations differs between sources and some of the guidelines are so general as to allow several forms of treatment. For example, most guidelines recommend neck dissection for lymph nodes that are clinically negative but where there is a high probability of lymph node metastases. How the probability of metastases is determined and the type of neck dissection recommended vary in the guidelines, some of which also recommend radiotherapy as an alternative or adjunct to neck dissection.

The aims of the study were to describe current patterns of treatment in three large areas of the UK, to investigate whether and how the treatment varies from guidelines and to investigate the influence of treatment on survival for UAT cancers.

### METHODS

This was a retrospective population-based multi-regional observational study. The Thames, West Midlands and Yorkshire cancer registries provided data on all malignant cases of UAT cancers (C00–C14, C30–C32) diagnosed and registered in their regions from 1984 to 1993. This comprised 18 795 cases, 44% of all cases of UAT cancers in England and Wales over his 10-year period (Office for National Statistics, cancer registration data). The regions have been anonymized at the request of the cancer registries.

Demographic information was available on patients' age at diagnosis, gender, deprivation, ethnic and marital status. We allocated a Carstairs deprivation score to each person based on their enumeration district of residence, linked through their postcode (Dolk et al, 1995). This is a well recognized measure of material deprivation and has been used in previous studies of UAT cancers (Thorne, 1997). Details of the tumour site, histological grade and extent of spread was available. TNM stage was available for only a minority of cases, however, a cancer registry extent of spread classification was available for regions 1 and 2. This classified tumours as within their organ of origin (level 1), having local spread (level 2), having nodal spread (level 3) and having distant metastases (level 4). Death was the only outcome measure available. The date and sequence of surgery, radiotherapy and chemotherapy was available for each case, but not details of the procedures, techniques or drugs used.

Six per cent of cases had information from death certificates only, ranging from 0.5% to 11% by region and another 6% of patients had had no active treatment. Treatment information was available for 13 510 patients being 77% complete. Treatment information was collected for the first 6 months following diagnosis in regions 1 and 2 and most treatments for the first 9 weeks following diagnosis in region 3.

We analysed crude survival (from all causes of death) from the date of diagnosis to the date of death or until 31 December 1995 for regions 1 and 2. Extent of spread and grade were not available

 *© 1999 Cancer Research Campaign*

**Table 2** Demographic and tumour characteristics of patients receiving treatment by region

| Number/% of patients | | Region 1 | | Region 2 | | Region 3 | |
|---|---|---|---|---|---|---|---|
| | | Number | % | Number | % | Number | % |
| Age at diagnosis | <60 years | 2127 | 30.7 | 1072 | 33.6 | 1063 | 31.4 |
| | 60–74 years | 3158 | 45.6 | 1483 | 46.5 | 1639 | 48.3 |
| | 75+ years | 1641 | 23.7 | 637 | 20.0 | 688 | 20.3 |
| Gender | Male | 4786 | 69.1 | 2201 | 68.9 | 2380 | 70.2 |
| UK Carstairs quintile | | | | | | | |
| | Quintile 1 | 1609 | 23.2 | 520 | 16.3 | 545 | 16.1 |
| | Quintile 2–3 | 3033 | 43.8 | 1134 | 35.5 | 1405 | 41.4 |
| | Quintile 4–5 | 2284 | 33.0 | 1540 | 48.2 | 1440 | 42.5 |
| Site distribution | | | | | | | |
| Mouth cancers C01–C06 | | 1792 | 25.9 | 1267 | 39.7 | 988 | 29.1 |
| Larynx C32 | | 2456 | 35.5 | 795 | 24.9 | 1263 | 37.3 |
| Pharynx excluding nasopharynx C09,C10,C12–C14 | | 21712 | 24.7 | 697 | 21.8 | 687 | 20.3 |
| Others | | 1517 | 13.9 | 690 | 10.7 | 566 | 13.5 |
| Registry Stage distribution | | | | | | | |
| Stage 1 (confined to organ of origin) | | 3011 | 47.1 | 1166 | 51.6 | Data missing | |
| Levels 2–4 | | 3383 | 52.9 | 1095 | 48.4 | Data missing | |

**Table 3** Treatment by site and age at diagnosis

| | | 75+ years | | 60–74 years | | <60 years | | Total | |
|---|---|---|---|---|---|---|---|---|---|
| | | Number | % | Number | % | Number | % | Number | % |
| Mouth C01–C06 | Radiotherapy | 544 | 51.3 | 841 | 42.7 | 581 | 38.1 | 1966 | 43.2 |
| | Surgery | 275 | 25.9 | 502 | 25.5 | 388 | 25.4 | 1165 | 25.6 |
| | Combined S&R* | 127 | 12.0 | 329 | 16.7 | 281 | 18.4 | 737 | 16.2 |
| | S&R* sequence | 114 | 10.8 | 298 | 15.1 | 276 | 18.1 | 688 | 15.1 |
| | Total | 1060 | 100 | 1970 | 100 | 1526 | 100 | 4556 | 100 |
| Pharynx C09, C10, C12–C14 | Radiotherapy | 483 | 64.0 | 870 | 56.5 | 558 | 49.3 | 1911 | 55.8 |
| | Surgery | 111 | 14.7 | 176 | 11.4 | 150 | 13.3 | 437 | 12.8 |
| | Combined S&R | 120 | 15.9 | 317 | 20.6 | 243 | 21.5 | 680 | 19.8 |
| | S&R sequence | 41 | 5.4 | 176 | 11.4 | 181 | 16.0 | 398 | 11.6 |
| | Total | 755 | 100 | 1539 | 100 | 1132 | 100 | 3426 | 100 |
| Larynx C32 | Radiotherapy | 714 | 73.1 | 1822 | 67.0 | 860 | 66.6 | 3396 | 68.1 |
| | Surgery | 91 | 9.3 | 260 | 9.6 | 112 | 8.7 | 463 | 9.3 |
| | Combined S&R | 121 | 12.4 | 405 | 14.9 | 198 | 15.3 | 724 | 14.5 |
| | S&R sequence | 51 | 5.2 | 234 | 8.6 | 122 | 9.4 | 407 | 8.2 |
| | Total | 977 | 100 | 2721 | 100 | 1292 | 100 | 4990 | 100 |

S = surgery; R = radiotherapy.

for region 3, so these cases were excluded from the survival analysis. Although the Office for National Statistics had not informed cancer registries of non-cancer deaths from 1992 onwards analysis of survival at different time periods showed that this did not affect the results. We used the SPSS package for analysis with a chi-squared test to assess differences in treatment. We used forward step-wise Cox proportional hazard model with a likelihood ratio test in the multivariate analyses. We undertook this for each site group, mouth (C01–C06), larynx (C32) and pharynx (C09, C10, C12–C14) entering the variables in the order: extent of spread, site (for mouth and pharynx, tumour grade, age, deprivation, marital status and treatment. All other sites were excluded from the survival analysis.

### RESULTS

The 12 972 patients had a total of 21 197 treatments with some patients having multiple treatments and some recurrences. Patients in region 1 were more affluent than in other regions. Region 2 had a different site distribution than other regions. This was due to incompleteness of treatment data for laryngeal cancers in this region (Table 2). There were no regional differences in incidence. The main treatments were radiotherapy and surgery. There were no major trends in treatment over time in any of the regions. Men with oral cancer were slightly more likely to have radiotherapy alone than women (46% compared with 40% $P < 0.001$) and women with oral cancer were more likely to receive surgery alone than men (30% compared with 23%, $P < 0.001$). There were no major gender differences in treatment of cancer of the larynx or pharynx.

There were some age-related differences in treatment, when site was taken into account. People over the age of 75 years at diagnosis were more likely to have had radiotherapy alone at all sites and less likely to have had surgery and radiotherapy in sequence (more than three months apart, $P < 0.001$, Table 3). Seven per cent of over 75-year-olds had no treatment compared with 2.5% of those less than 75 years of age ($P < 0.001$).

© 1999 Cancer Research Campaign

**Table 4** Timing and order of treatments for people who had both radiotherapy and surgery

| Number (%) | Region 1 1984–1992 | | Region 2 1984–1993 | | Region 3 1984–1993 | |
|---|---|---|---|---|---|---|
| | Number | % | Number | % | Number | % |
| Total number of patients having both surgery and radiotherapy | 7485 | 50.0 | 1481 | 23 | 435 | 15.7 |
| Treatments in sequence (more than 3 months between treatments) | | | | | | |
| Surgery followed by radiotherapy | 1222 | 16.3 | 440 | 32.7 | 95 | 15.0 |
| Radiotherapy followed by surgery | 1108 | 14.8 | 355 | 26.4 | 59 | 9.0 |
| Combined treatment (within 3 months) | 5155 | 68.9 | 552 | 41.0 | 496 | 76.0 |
| Surgery first | 4361 | 84.6 | 477 | 86.4 | 435 | 87.7 |
| Radiotherapy first | 403 | 7.8 | 59 | 10.7 | 7 | 1.4 |
| Within 1 week | 391 | 7.6 | 16 | 2.9 | 54 | 10.9 |
| Combined treatments within 1 month | 2122 | 40.9 | 143 | 25.9 | 131 | 45.4 |
| Combined treatment 1–3 months apart | 3043 | 59.1 | 409 | 74.1 | 157 | 54.6 |

**Table 5** Multivariate analysis of crude survival by treatment. Site, stage, grade, age, deprivation and marital status in Cox's proportional hazard model.

| Site number of cases | Mouth 2222 | | Larynx 2648 | | Pharynx 1865 | |
|---|---|---|---|---|---|---|
| Risk of death relative to Radiotherapy alone | Relative risk | 95% CI | Relative risk | 95% CI | Relative risk | 95% CI |
| Surgery alone | 0.46 | 0.39–0.53 | 1.52 | 1.29–1.81 | 0.86 | 0.71–1.03 |
| Radiotherapy & surgery combined (<3 months apart) | 0.64 | 0.55–0.74 | 1.15 | 1.00–1.33 | 0.66 | 0.57–0.76 |
| Radiotherapy & surgery in sequence (>3+ months apart) | 0.80 | 0.69–0.92 | 1.32 | 1.12–1.58 | 0.80 | 0.68–0.95 |
| Risk of death relative to S&R in combination | | | | | | |
| Radiotherapy & surgery in sequence (>3+ months apart) | 1.25 | 1.04–1.49 | 1.15 | 0.94–1.41 | 1.19 | 1.07–1.31 |

The recommended treatments outlined in Table 1 were adhered to in 60% of all treatments. Those guidelines that allowed for several forms of treatment had higher adherence as would be expected (Table 1). Cancer registry levels 2–4 are broadly equivalent to T3 or T4 stages in that a tumour greater than 4 cm in the upper aerodigestive tract (T3 or above) is likely to invade adjacent structures (level 2 or above) (Howells, 1995). Cancer registry level 1 is equivalent to T1 and T2. Some patients at cancer registry level 1 had combination therapy accounting for the low adherence to guidelines. Only 38% of patients with salivary gland cancers had the recommended treatment of surgery alone for extent of spread 1 tumours, whereas 85% had surgery either alone or in combination with radiotherapy. Similarly, only 35% of patients had the recommended treatment of both surgery and radiotherapy for level 2–4 oral cancers; 42% had radiotherapy alone and another 23% surgery alone. In this analysis the timing and order of treatment was not taken into account. If it had been, the adherence to guidelines may have been lower.

Thirty per cent of all patients had both surgery and radiotherapy; 32% of patients in region 1, 29% in region 2 and 16% in region 3, treatment details being recorded for the first 26 weeks in regions 1 and 2 and for the first 9 weeks in region 3 (Table 4). There was a trend towards dual treatment with 20% of patients treated in 1984–1986 having both surgery and radiotherapy compared with 29% in 1987–1990 and 30% in 1991–1993 ($P < 0.001$). Preoperative radiotherapy comprised 7.5% of combined treatment and this did not vary much by site or time. Fewer than half of the patients treated with combined surgery and radiotherapy (within 3 months) had the treatments less than 1 month apart, as currently recommended (Glaholm 1998), ranging from 25% for region 2 to 45% for region 3. A greater proportion of patients treated with both surgery and radiotherapy had their treatments within 3 months of each other over time (68% in 1984–1986, 76% in 1987–1990 and 84% in 1991–1993 $P < 0.001$) but there was no increase in the proportion of patients who had the treatments within 1 month of each other.

Oral cancer patients who were treated by surgery had better survival than those treated with radiotherapy, or both surgery and radiotherapy. Oral cancer patients treated with surgery alone had half the risk of death (relative risk (RR) 0.46, 0.39–0.53), and those with combined treatment two-thirds the risk of death (RR 0.64, 0.55–0.74) of those who had radiotherapy alone (Table 5). These differences occurred independent of tumour and demographic prognostic factors. Although either radiotherapy or surgery alone was recommended in the guidelines for small mouth cancers (T1/T2, Table 1), oral cancers confined to their organ of origin treated by surgery had significantly better survival than radiotherapy (Figure 1).

Radiotherapy was associated with better survival for cancer of the larynx compared with surgery or surgery and radiotherapy in sequence independent of tumour or demographic factors (Table 5). Combined treatment was independently associated with the best

*© 1999 Cancer Research Campaign*

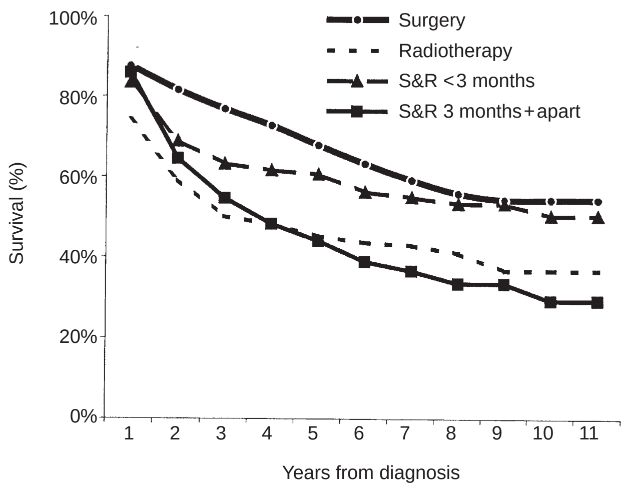

**Figure 1** Survival of patients with mouth cancers (C01–C06) confined to organ of origin by treatment survival for pharyngeal cancers with no significant differences between surgery and radiotherapy alone (Table 5).

Combined surgery and radiotherapy (within 3 months) was associated with significantly better survival independent of tumour and demographic factors for oral and pharyngeal cancers compared with surgery and radiotherapy treatment in sequence (more than 3 months apart, Table 5). All the survival differences were most pronounced when prognosis was good (data not shown). The differences in survival demonstrated were similar for both crude and cause specific survival.

### DISCUSSION

This is the first population-based large case series of UAT cancer treatment in the UK. Although the study is limited by the uniformity and completeness of the data it provides useful information on treatment. The treatments given differ from those currently recommended. This may be because the evidence for some recommendations such as the benefits of post- rather than preoperative radiotherapy was published in 1991 (Tupchong, 1991), after the majority of the patients in this study were treated. This study provides baseline information on treatment patterns. A future population-based analysis of the UAT cancer treatment could audit the effectiveness of the current guidelines. Both improved comparability of treatment information between cancer registries and the implementation of a national audit dataset for UAT cancers would facilitate any future studies in this area.

The study highlights treatment differences by geographical area and patient age and gender. Stell (1990) found that much of the difference in survival by age group was accounted for by the proportion not treated.

This was a retrospective observational study and because of this the results of the survival analysis are less conclusive than a prospective randomized controlled trial. Differences in recording of treatment details between registries, the lack of TNM staging and the incompleteness of the data limited the analysis. Although the registry stage 1 is broadly equivalent to T1 and T2 this may not have been so for a minority of cases. The details of surgical procedures or radiotherapy doses are not available and so comparisons between different surgical or radiotherapy techniques are not possible. For example, some of the people receiving both surgery and radiotherapy may have had radiotherapy to the primary lesion and a surgical neck dissection, or vice versa, rather than true combined therapy to the primary tumour. Survival is only one outcome among many and no information is available on function or quality of life. As there are relatively few randomized controlled trials in this area this study does provide some information on a large population group and raises questions as to the effect of different treatments on survival.

Survival was associated with the content of treatment. There are many possible reasons for this. Potential biases include the proportion of missing data, data accuracy and selection biases in treatments. Although tumour site, extent of spread, tumour grade and patient age and deprivation were taken into account in the model there may have been clinical differences between treatment groups that were not accounted for in the model. In order to find out if the treatments cause the differences in survival greater involvement in prospective large randomized trials or multi-centre audits would be needed.

Oral cancer patients who had surgery had better survival than patients who had combination treatment or radiotherapy. Although surgery was associated with better survival for small oral cancers (level 1, within organ of origin), either surgery or radiotherapy is recommended for small oral cancers and only 33% of patients with small oral cancers had surgery whilst 38% had radiotherapy. This contrasts with the situation for advanced oral cancers where combination therapy is of proven benefit (Mendenhall et al, 1988; Weber et al, 1990). Nisi (1998) found that patients with advanced tongue cancer treated by combination treatment had better local control but no better survival than patients treated by surgery alone. As patients who have combination treatment for oral cancer have been shown to have worse quality of life than patients having surgery alone (Finlay, 1984; Rathmell, 1991) the rationale for combination treatment for T1 and T2 tumours may need to be examined.

The better survival for patients with cancer of the larynx treated by radiotherapy supports the findings of other studies (Vermund, 1990) and is in line with current recommendations (Table 1). Combined treatment was associated with the best survival for pharyngeal cancers as is recommended for T3 and T4 cancers.

The timing of treatments was associated with survival. Oral cancer patients who had combined surgery and radiotherapy (within 3 months) had better survival than those who had both surgery and radiotherapy in sequence (3 months or more apart). There are several possible explanations for this survival difference. The delays may occur in patients who are physically unable to undergo combination treatments due to their general condition; the subsequent treatment may be for a recurrence; the treatment may be palliative and episodic; or delays in treatment may allow tumour repopulation and decrease survival as has been found in other studies of UAT cancers (Amdur et al, 1989; Skaladowski et al, 1994; Duncan et al, 1996; Wang et al, 1996). Appropriate timing of treatments may improve survival of patients with UAT cancers.

This study does not provide conclusive evidence that treatment modality affects survival from cancers of the mouth, pharynx or larynx. It does raise a number of questions. How well are treatment protocols being followed? Does surgery produce better results than radiotherapy for T1 and T2 tumours of the mouth in terms of

© 1999 Cancer Research Campaign survival, function and quality of life once detailed clinical factors have been accounted for? Are there avoidable delays between different modalities in combination therapy that affect survival? The answers to these questions need to be investigated through large randomized controlled trials and service audits that include functional and quality of life components and systematic reviews of randomized controlled trials.

### ACKNOWLEDGEMENTS

We would like to thank and acknowledge the support from the individuals and organizations involved in this collaborative study. They include Karen Linklater and Jan Bell from the Thames Cancer Registry, Emma Lees and Lillian Sommervaille from the West Midlands Cancer Registry, Michael Shires and Lesley Rider from the Yorkshire Cancer Registry, Mike Quinn and Jennifer Jones from the Office for National Statistics and Michel Coleman from the London School of Hygiene and Tropical Medicine. We also thank the United Kingdom Co-ordinating Committee on Cancer Research for providing details of clinical trials that recruited patients during the period of the study.

### REFERENCES

Amdur RJ, Parsons JT, Mendenhall MW, Million RR and Cassisi NJ (1989) Split course versus continuous-course irradiation in the postoperative setting for squamous cell carcinoma of the head and neck. *Int J Radiat Oncol Biol Phys* **17**: 279–285

American Society for Head and Neck Surgery and The Society of Head and Neck Surgeons (1996) *Clinical Practice Guidelines for the Diagnosis and Management of Cancer of the Head and Neck*

Bentzen SM, Johansen LV and Overgaard J (1991) Clinical radiobiology of squamous cell carcinoma of the oropharynx. *Int J Rad Oncol Biol Phy* **20**: 1197–1206

Berrino F, Sant M, Verdicchia A, Capocaccia R, Hakulinen T and Esteve J (1995) *Survival of Cancer Patients in Europe: the EUROCARE Study*. IARC Scientific Publication No 132. IARC: Lyon

British Association of Head and Neck Oncologists (1998) Provision and quality assurance for head and neck cancer care in the United Kingdom: a nationally co-ordinated multidisciplinary approach.

British Association of Otolaryngologists–Head and Neck Surgeons (1998) *Effective Head and Neck Cancer Management: Consensus Document*.

Brown AE and Langdon JD (1995) Management of oral cancer. *Ann Royal Coll Surg Engl* **777**: 404–408

Christensen E (1987) Individual and therapy dependent prognosis based on data from controlled clinical trials in chronic liver disease. PhD thesis. Copenhagen University: Copenhagen

Davidson J, Boyd B, Gulland P et al (1991) A comparison of the results following oromandibular reconstruction using a radial forearm flap with either radial bone or a reconstruction plate. *Plast Reconstr Surg* **88**: 201–208

Dolk H, Mertens B, Kleinschmidt I, Walls P, Shaddick G and Elliot P (1995) A standardised approach to the control socioeconomic confounding in small area studies or environment and health. *J Epidemiol Comm Health* **49**: S9–S24

Duncan W, MacDougal RH, Kerr GR and Downing D (1996) Adverse effects of treatment gaps in the outcome of radiotherapy for laryngeal cancer. *Radioth Oncol* **42**: 203–207

Edwards D, Johnson NW, Cooper D and Warnakulasuriya KAAS (1996) Management of cancers of the head and neck in the UK: questionnaire survey of consultants. *BMJ* **315**: 1589

Fein DA, Mendenhall WM, Parsons JT, et al (1994) Carcinoma of the oral tongue: a comparison of results and complications of treatment with radiotherapy and/or surgery. *Head Neck* **16**: 358–365

Finlay PM, Dawson F, Robertson AG and Soutar DS (1992) An evaluation of functional outcome after surgery and radiotherapy for intraoral cancer. *Br J Oral Maxillofac Surg* **30**: 14–17

Glaholm J (1999) Oral cancer; the role of radiotherapy and chemotherapy. In: *A Textbook of Oral Cancer*. Shah J, Batasakis J and Johnson NW (eds). ISIS Medical: Oxford

Grimshaw J and Russell I (1993) Achieving health gain through clinical guidelines. Developing scientifically valid guidelines. *Quality Health Care* **31**: 552–558

Harrison LB, Zelefsky MJ, Armstrong JG, Carper E, Gaynor JJ and Sessions RB (1994) Performance status after treatment for squamous cell cancer of the base of tongue – a comparison of primary radiation therapy versus primary surgery. *Int J Radiat Oncol Biol Phys* **30**: 953–957

Howells SE (1995) A comparison of the influence of two different staging classification on five year survival for oral cancer. MSc. London: University of London

MacFarlane GJ, Boyle P and Scully C (1992) Oral cancer in Scotland: changing incidence and mortality. *Br Med J* **305**: 1121–1123

Mendenhall WM, Parsons JT, Stringer SP, Cassisi NJ and Million RR (1988) T1–T2 vocal cord carcinoma: a basis for comparing the results of radiotherapy and surgery. *Head Neck Surg* **10**: 373–377

Mitchell R and Crighton LE (1993) The management of patients with carcinoma of the tongue. *Br J Oral Maxillofac Surg* **31**: 304–308

Moore GJ, Parsons JT and Mendenhall WM (1996) Quality of life outcomes after primary radiotherapy for squamous cell carcinoma of the base of the tongue. *Int J Radiat Oncol Biol Phys* **36**: 351–354

Morton RP, Davies AD, Baker J, Baker GA and Stell PM (1984) Quality of life in treated head and neck cancer patients: a preliminary report. *Clin Otolaryngol* **9**: 181–185

Morton RP (1997) Laryngeal cancer: quality of life and cost effectiveness. *Head Neck* **19**: 243–250

Nisi KW, Foote RL, Bonner JA and McCaffrey TV (1998) Adjuvant radiotherapy for squamous cell carcinoma of the tongue base: improved local-regional disease control compared with surgery alone. *Int J Radiat Oncol Biol Phys* **41**: 371–377

O'Hanlon S, Forester DP and Lowry J (1997) Oral cancer in the North East of England: incidence, mortality trends and the link with material deprivation. *Comm Dent Oral Epidemiol* **25**: 371–376

Parkin DM, Pisani P and Ferlay J (1993) Estimates of the worldwide incidence of eighteen major cancers in 1985. *Int J Cancer* **54**: 594–606

Platz H, Froes R and Hudec MA (1986) Prognoses of Oral Cavity Carcinomas; results of a multicentric retrospective observational study. Munchen, Wein: Hanser

Priestman TJ, Bullimore JA, Godden TP and Ceusch GP (1989) The Royal College of Radiologists Fractionation Survey. *Clin Oncol* **1**: 39–46

Rathmell AJ, Ash DV, Howes M and Nicholls J (1991) Assessing quality of life in patients treated for advanced head and neck cancer. *Clin Oncol* **3**: 10–16

Skaladowski K, Law MG. Maciejejewski B and Steel GG (1994) Planned and unplanned gaps in radiotherapy; the importance of gap position and gap duration. *Radioth Oncol* **30**: 109–120

Soutar CS and McGregor D (1986) The radial forearm flap in intraoral reconstruction. The experience of 60 consecutive cases. *Plas Reconstr Surg* **78**: 1–8

Stell PM (1990) Prognosis in laryngeal carcinoma: host factors. *Clin Otolaryngol* **15**: 111–119

Stewart MG, Chen AY and Stach CB (1998) Outcomes of voice and quality of life in patients with laryngeal cancer. *Arch Otolaryngol Head Neck Surg* **124**: 143–148

TonVan J, Lefebvre JL, Stern JC, Buisset E, Coche-Dequeant B and Vankemmel B (1991) Comparison of surgery and radiotherapy in T1 and T2 glottic carcinomas. *Am J Surg* **162**: 337–340

Thorne P, Etherington D and Birchall MA (1997) Head and neck cancer in the South West of England: influence of socio-economic status on incidence and second primary tumours. *Eur J Surg Oncol* **23**: 503–508

Tupchong L (1991) Pre-op versus post-op radiotherapy for head and neck squamous carcinoma, final results of trial RTOG 73–03. *Int J Radiat Biol Phys* **20**: 21–28

Vaughan ED, Bainton R and Martin IC (1992) Improvements in morbidity of oral cancer using microvascular free flap reconstructions. *J Craniomaxillofac Surg* **20**: 132–134

Vaughan ED and Brown JS (1994) The management of the mandible in mouth cancer. *Br J Oral Maxillofac Surg* **32**: 345–346

Vermund H, Boysen M, Brandenbug JH, Evensen J, Jacobsen AB, Kalljus O et al (1990) Primary irradiation, surgery or combined therapy in squamous cell carcinoma of the larynx. A comparison of treatment results from two centres. *Acta Oncol* **29**: 489–503

Wang CC, Efird J, Nakfoor B and Martins P (1996) Local control of T3 carcinomas after accelerated fractionation; a look at the gap. *Int J Radiat Oncol Biol Phys* **35**: 439–441

Weber RS, Gidley P, Morrison WH, Peters LJ, Hankins P, Wolf P and Gulliamondegui O (1990) Treatment selection for carcinoma of the base of the tongue. *Am J Surg* **160**: 415–419

© 1999 Cancer Research Campaign

Woolgar JA, Brown JS, Scott J, West CR, Vaughan ED and Rogers S (1995) Survival, metastasis and recurrence of oral cancer in relation to pathological features. *J Royal Coll Surg Engl* **77**: 325–331

Worrall SF and Corrigan M (1995) An audit of oral squamous cell carcinoma using a computerised malignancy database. *Ann R Coll Surg Engl* **77**: 332–336

Zelefsky MJ, Gaynor JJ, Kraus D, Strong EW, Shah JP and Harrison LB (1996) Long-term subjective functional outcome of surgery plus postoperative radiotherapy for advanced stage oral cavity and oropharyngeal carcinoma. *Am J Surg* **171**: 258–261

© 1999 Cancer Research Campaign 
